# Involvement of Bile Acid Metabolism and Gut Microbiota in the Amelioration of Experimental Metabolism-Associated Fatty Liver Disease by Nobiletin

**DOI:** 10.3390/molecules29050976

**Published:** 2024-02-23

**Authors:** Hongling Xu, Mingming Yuan, Kailin Niu, Wei Yang, Maoyuan Jiang, Lei Zhang, Jing Zhou

**Affiliations:** 1 School of Traditional Chinese Pharmacology, Chengdu University of Traditional Chinese Medicine, Chengdu 611137, China; xuhongling@stu.cdutcm.edu.cn (H.X.); niukailin2022@163.com (K.N.); 2 Laboratory Animal Center Affiliate from Research Office, Sichuan Academy of Chinese Medicine Sciences, Chengdu 610041, China; ymm-wq@163.com (M.Y.); yangwei20231211@163.com (W.Y.); 3 State Key Laboratory of Quality Research in Chinese Medicine, Institute of Chinese Medical Sciences, University of Macau, Macau 999078, China; maoyuanjiangsoc@126.com

**Keywords:** nobiletin, bile acid, gut microbiota, farnesoid X receptor, metabolism-associated fatty liver disease

## Abstract

Metabolism-associated fatty liver disease (MAFLD), a growing health problem worldwide, is one of the major risks for the development of cirrhosis and liver cancer. Oral administration of nobiletin (NOB), a natural citrus flavonoid, modulates the gut microbes and their metabolites in mice. In the present study, we established a mouse model of MAFLD by subjecting mice to a high-fat diet (HFD) for 12 weeks. Throughout this timeframe, NOB was administered to investigate its potential benefits on gut microbial balance and bile acid (BA) metabolism using various techniques, including 16S rRNA sequencing, targeted metabolomics of BA, and biological assays. NOB effectively slowed the progression of MAFLD by reducing serum lipid levels, blood glucose levels, LPS levels, and hepatic IL-1β and TNF-α levels. Furthermore, NOB reinstated diversity within the gut microbial community, increasing the population of bacteria that produce bile salt hydrolase (BSH) to enhance BA excretion. By exploring further, we found NOB downregulated hepatic expression of the farnesoid X receptor (*FXR*) and its associated small heterodimer partner (*SHP*), and it increased the expression of downstream enzymes, including cholesterol 7α-hydroxylase (*CYP7A1*) and cytochrome P450 27A1 (*CYP27A1*). This acceleration in cholesterol conversion within the liver contributes to mitigating MAFLD. The present findings underscore the significant role of NOB in regulating gut microbial balance and BA metabolism, revealing that long-term intake of NOB plays beneficial roles in the prevention or intervention of MAFLD.

## 1. Introduction

With the increasing prevalence of obesity and metabolic syndrome, metabolism-associated fatty liver disease (MAFLD) is overtaking viral hepatitis as the most common chronic liver disease worldwide. MAFLD currently affects approximately 25% of the global population [1]. MAFLD leads to liver-related conditions, such as cirrhosis and hepatocellular carcinoma, and it increases mortality rates associated with liver diseases. In addition, MAFLD is closely linked to a high prevalence of type 2 diabetes mellitus, arteriosclerotic cardiovascular diseases, cerebral disorders, renal issues, vascular complications, and extraneous malignancies [2]. Therefore, timely and effective management of MAFLD is essential to halt the progression of liver diseases and address the prevention and treatment of associated metabolic and cardiovascular risk factors and complications. Unfortunately, no specific drugs have been designed for MAFLD [3], thus making lifestyle interventions the cornerstone of MAFLD management [4], which has prompted intense research into more sustainable treatments for this condition.

In addition to supplying the body with essential metabolites for health [5], gut microbes also have an impact on fat absorption [6]. Gut microbes play a significant role in promoting the liver utilization of cholesterol for synthesizing bile acid (BA) by regulating the expression of key enzymes and regulatory factors in lipid metabolism. This regulation results in the conversion or degradation of cholesterol, effectively lowering its levels in the body. Thus, gut microbes modulate blood lipid levels by influencing cholesterol metabolism, primarily by inhibiting cholesterol synthesis and facilitating its degradation and conversion. As research in the field of intestinal microecology advances, an increasing number of studies have demonstrated the significance of gut microbiota disorders, small intestinal bacterial overgrowth, and intestinal-borne endotoxemia resulting from bacterial translocation in the pathogenesis of MAFLD. Moreover, restoring and adjusting the balance of the intestinal microecosystem may play a crucial role in slowing the progression of the disease and reducing the occurrence of cirrhosis and its associated complications [7]. Alterations in the composition and function of gut microbes affect MAFLD through the production of various metabolites, which, in turn, impact serum cholesterol levels [8].

BAs are important in lipid metabolism because they emulsify lipids, increase their interaction with lipase, and regulate the activity of pancreatic lipase and lipoprotein esterase. Consequently, BA enhances lipid hydrolysis metabolism in vivo [9]. BA produced by cholesterol catabolism facilitates the digestion and absorption of dietary lipids and actively participates in the regulation of lipid metabolism [10]. Several studies have demonstrated that alterations in gut microbiota composition influence the BA profile of the host [11,12,13], and BSH-producing gut bacteria hydrolyses conjugated BAs into unconjugated forms, further converting them into secondary BAs. *FXR* is an endogenous bile acid-activated nuclear hormone receptor that is highly expressed in hepatocytes. *FXR* controls all aspects of BA metabolism, including synthesis, transport to bile ducts, and intestinal absorption [14]. Additionally, the *FXR* undergoes feedback regulation by BA [15]. In mouse models, taurine-β-cholic acid (T-βMCA) is a prominent primary BA that has emerged as a potent natural *FXR* antagonist. T-βMCA-mediated inhibition of intestinal *FXR* expression mitigates non-alcoholic fatty liver disease (NAFLD) induced by a high-fat diet [16]. Various deoxycholic acids, such as tauroursodeoxycholic acid (TUDCA) and taurochenodeoxycholic acid (TCDCA), have been demonstrated to reduce *FXR* expression [17]. *FXR* activation may alleviate NAFLD by reducing hepatic lipid levels and intestinal lipid absorption [14]. Hepatic *FXR* activation induces the transcription of *SHP* and regulates cholesterol metabolism by inhibiting *CYP7A1* [18].

Nobiletin (NOB), a flavonoid derived from citrus, exhibits therapeutic potential for various diseases, including asthma, colitis, and Alzheimer’s disease [19,20,21]. NOB provides hepatoprotective effects by regulating the composition of gut microbes [22] and simultaneously mitigating insulin resistance [23]. Studies have demonstrated that NOB can ameliorate hepatic lipid deposition, oxidative stress, and inflammation through mechanisms involving the Nrf2/NF-κB axis in NAFLD [24]. Additionally, NOB has been shown to have beneficial effects in mice deficient dyslipidemia in hepatic AMPK deficient (*Ampkβ*1^−/−^*)* mice fed a high-fat diet (HFD) [25]. These regulatory effects may help explain the beneficial activity of NOB in reducing hepatic fat deposition and ameliorating metabolic disorders. However, it remains unclear whether the effects of NOB on MAFLD in mice are associated with gut microbial balance and BA metabolism.

Here, we examined the impact of nobiletin on the advancement of MAFLD in mice and explored whether it acts via modulation of gut microbial balance and BA metabolism. Our study aims to provide direct evidence of its efficacy and delve deeper into its mechanism of action. We aim for this study to offer fresh ideas and insights for the clinical prevention and treatment of MAFLD.

## 2. Results

### 2.1. NOB Treatment Attenuated HFD-Induced Weight Gain and Insulin Resistance (IR) in Mice

**Figure 1:**
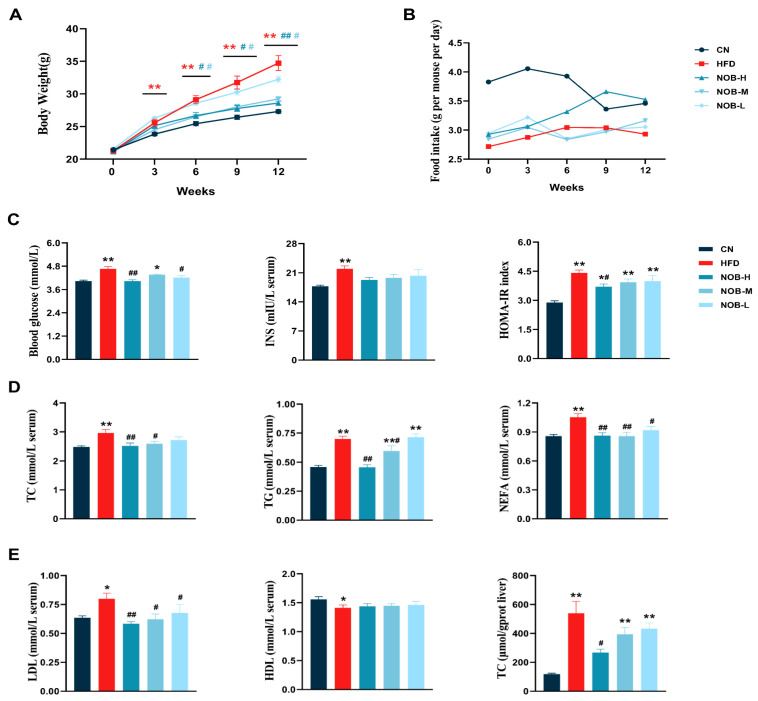
NOB improves dyslipidemia and insulin resistance in HFD-fed mice. (**A**) The dynamic changes in body weight. (**B**) The dynamic changes in Food intake. (**C**) Fasting blood glucose, serum insulin levels, and HOMA-IR index. (**D**) Serum contents of TC, TG, and NEFA. (**E**) Serum LDL levels, serum HDL levels, and liver TC levels. * *p* < 0.05, ** *p* < 0.01 compared to the CN group; # *p* < 0.05, ## *p* < 0.01 compared to the HFD group (ANOVA, *n* = 6 each group, mean ± SEM).

The HFD group exhibited a significant increase in body weight compared to the CN group. The weight gain became evident at Week 3 and increased over time. Daily oral administration of NOB led to a reduction in body weight among HFD-fed mice. Various doses of NOB (10, 50, 100 mg/kg) were associated with significant reductions in body weight at different time points (Figure 1A). A significant difference in body weight between the NOB-H treated groups and the HFD group was observed from Week 6 and increased over time.

Regarding changes in food intake (Figure 1B), the highest food consumption was observed in Week 3, and a higher intake was observed in the NOB-H group compared to the HFD, HFD + NOB-M, and HFD + NOB-L groups. Because MAFLD is closely correlated with glucose tolerance and insulin resistance (IR), the levels of glucose, insulin, and homeostatic model assessment–insulin resistance (HOMA-IR) index were evaluated. Compared to the HFD group, NOB treatment resulted in significantly lower levels of glucose, insulin, and HOMA-IR index (Figure 1C).

### 2.2. NOB Treatment Attenuates Lipid Levels in MAFLD Mice

The lipid profiles were assessed by measuring serum levels of key biochemical markers, including TC, TG, NEFA, LDL, HDL, as well as liver TC levels.

In the HFD group, there was a significant increase in TC, TG, NEFA, and LDL levels compared to the CN group, which were significantly mitigated after NOB intervention (Figure 1D). Furthermore, NOB treatment significantly decreases liver TC levels in HFD-fed mice (Figure 1E).

### 2.3. NOB Treatment Improves Liver Biochemical Indices and Inflammatory Factor Levels in MAFLD Mice

**Figure 2:**
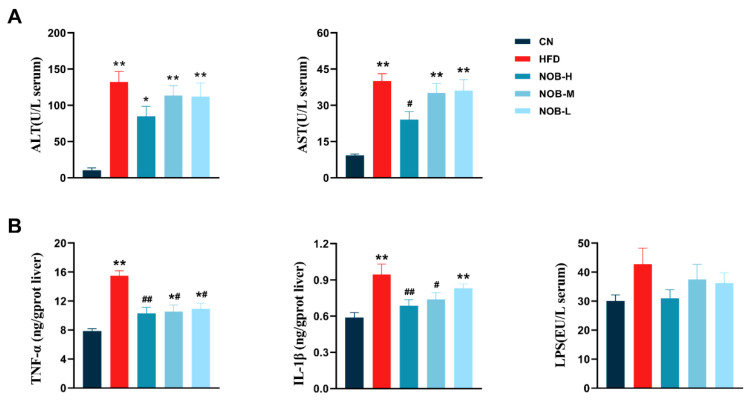

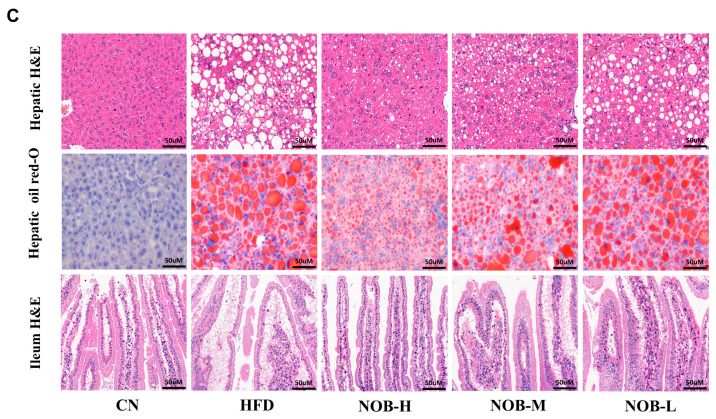
NOB reduces inflammatory and liver lipid droplet accumulation in HFD-fed mice. (**A**) Serum ALT and AST concentrations. (**B**) Liver TNF-α, IL-1β and LPS concentrations. * *p* < 0.05, ** *p* < 0.01 compared to the CN group; # *p* < 0.05, ## *p* < 0.01 compared to the HFD group (ANOVA, *n* = 6 in each group, mean ± SEM). (**C**) Representative images of liver sections with H&E staining and Oil Red O staining. H&E staining of ileum sections (400×, scale bar = 50 am, *n* = 6–8 in each group).

The HFD group had significantly elevated ALT and AST levels compared to the CN group. However, the levels of ALT and AST in mice treated with NOB-H were reduced (Figure 2A).

Assessment of inflammatory factors in the liver indicated that the levels of TNF-α and IL-1β were significantly higher in the HFD group than in the CN group. In addition, the TNF-α and IL-1β levels were mitigated after NOB treatment. Moreover, there was no significant change in serum LPS levels among the groups (Figure 2B).

### 2.4. Effect of NOB Treatment on Histopathology of Liver and Ileum in MAFLD Mice

As shown in Figure 2C, H&E staining revealed that the liver lobules of mice in the CN group displayed a structurally regular pattern, with no fat accumulation in hepatocytes or infiltration of inflammatory cells. However, mice in the HFD group exhibited varying degrees of fatty vacuoles. Some of these livers showed minimal hepatocellular necrosis and limited inflammatory cell infiltration in the necrotic area and the surrounding hepatic sinusoids, as well as modest fibrous tissue proliferation. After the administration of NOB, the fatty alterations, inflammatory cell infiltration, and hepatocyte ballooning in the liver tissues of the mice were significantly reduced, indicating substantial improvement.

Oil Red O staining indicated a higher expression of adipose tissue in the livers of mice in the HFD group compared to the CN group, indicating severe liver fat accumulation after a 12-week HFD regimen. Hepatic lipid deposition was significantly ameliorated in the NOB-H-treated group compared to the HFD group.

The mouse ileum was observed using H&E staining, which indicated no significant changes in the mucosal, submucosal, muscular, and outer membrane structures of the ileal tissues across all treatment groups. The mucosa was coated with a single layer of columnar epithelial cells, which were neatly arranged, and no inflammatory infiltration or fibroplasia was observed.

### 2.5. NOB Regulates BA Composition in the Feces of MAFLD Mice

**Figure 3:**
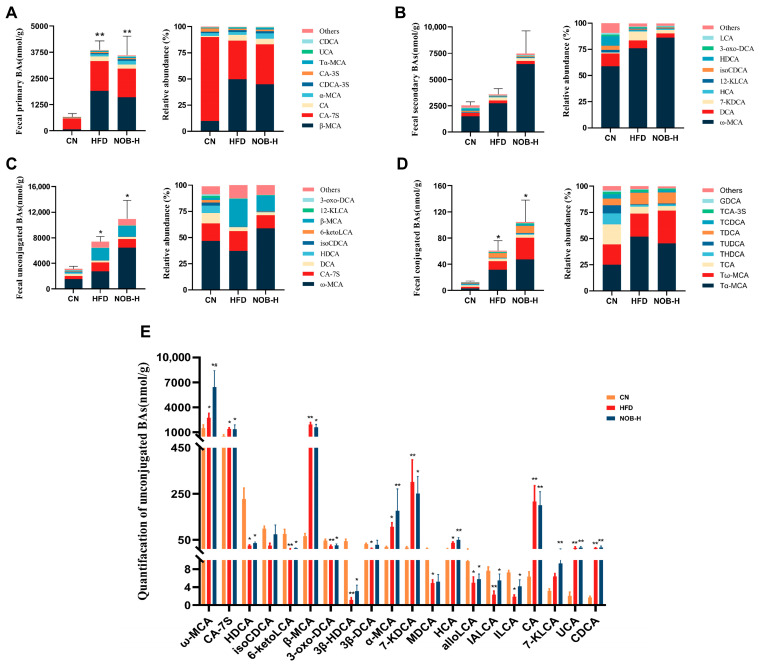
NOB alters BA profiles in HFD-fed mice. (**A**) Effects of NOB treatment on fecal primary BAs and composition (percentage). (**B**) Effects of NOB treatment on fecal secondary BAs and composition (percentage). (**C**) Effects of NOB treatment on fecal unconjugated BAs and composition (percentage). (**D**) Effects of NOB treatment on fecal conjugated BAs and composition (percentage). (**E**) Quantification of faecal unconjugated BAs. * *p* < 0.05 and ** *p* < 0.01 compared to the CN group; # *p* < 0.05 compared to the HFD group (ANOVA, *n* = 5 in each group, mean ± SEM).

To investigate whether NOB regulates BA metabolism in HFD-fed mice, the feces of five mice from each of the CN, HFD, and NOB-H groups of mice were randomly selected, and the feces’ BA content was quantified using ultra-performance liquid chromatography–tandem mass spectrometry (UPLC–MS/MS). A total of 65 BA species were tested and the feces BAs in each group were evaluated according to the content rankings of primary/secondary BAs and conjugated/unconjugated BAs (Figure 3).

The feces levels of primary/secondary BAs were elevated in both the HFD and NOB-H groups compared to the CN group (Figure 3A,B). NOB-H intervention adjusted the composition of primary BAs, leading to a normalization of the ratio of each type of BA, resembling mice in the CN group. Regarding secondary BAs, the HFD group of mice exhibited increased levels, and NOB-H intervention further heightened these secondary BA levels. Likewise, both conjugated and unconjugated BAs were already enriched in the HFD group, and the addition of NOB-H intervention further elevated fecal BA levels compared to the HFD group, the distribution of each type of BA was adjusted to bring it closer to that of the CN group. (Figure 3C,D).

In the HFD and NOB-H groups, the levels of ω-MCA, CA-7S, β-MCA, α-MCA, 7-KDCA, CA, UCA, CDCA, TαMCA, TMCA, TCA-3S, TβMCA, GCA, and GLCA-3S were increased, and the levels of 6-ketoLCA, 3-oxo-DCA, 3β-HDCA, 3β-DCA, MDCA, alloLCA, IALCA, and LCA were decreased compared to the CN group (Figure 3E). ω-MCA was the most abundant secondary and unconjugated BA in all three groups of mice tested, and it greatly augmented after NOB-H administration, showing significant differences compared to both the control and HFD groups (Figure 3B,E). The increase in the feces concentration of secondary BAs indicates enhanced BA excretion and increased hepatic BA secretion; this process promotes hepatic cholesterol depletion and reduces the accumulation of hepatic lipids [26].

### 2.6. NOB Alters the Composition of Gut Microbes in MAFLD Mice

**Figure 4:**
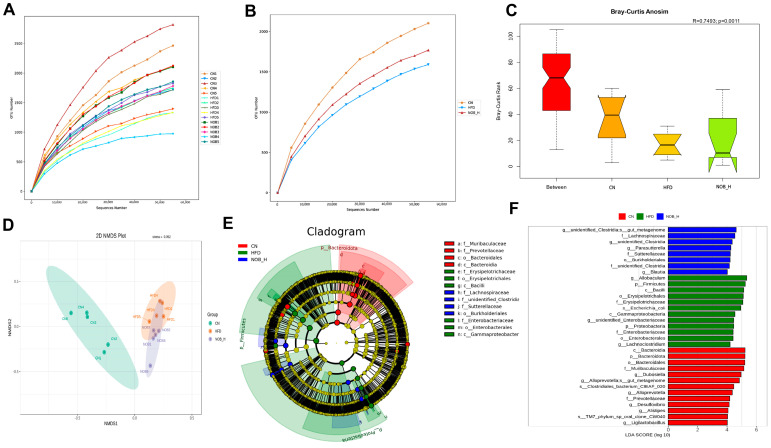

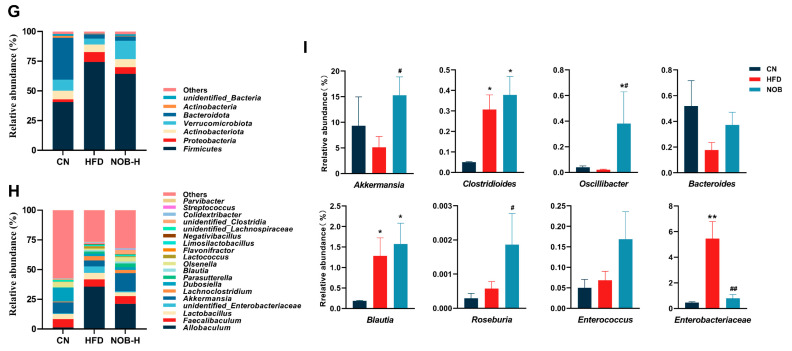
NOB alters the gut microbiota composition and increases BSH-producing microbes in HFD-fed mice. (**A**) Rarefaction curves of cecum content samples in individuals. (**B**) Rarefaction curves of cecum content samples in groups. (**C**) Weighted UniFrac ANOSIM analysis among the three groups. (**D**) Nonmetric multidimensional scaling (NMDS) analysis of each group. (**E**,**F**) Visualization of linear discriminant analysis effect size (LEfSe). (**G**) Relative abundance of gut microbiota at the phylum level. (**H**) Relative abundance of gut microbiota at the genus level. (**I**) Relative abundance of BSH-producing bacteria in each group. * *p* < 0.05, ** *p* < 0.01 compared to the CN group; # *p* < 0.05, ## *p* < 0.01 compared to the HFD group (ANOVA, *n* = 5 each group, mean ± SEM).

As mentioned earlier, the composition of BAs in feces underwent significant changes following HFD and NOB-H interventions. Considering that BA relies on the assistance of gut microbes for metabolism, our next step involved utilizing 16S rRNA sequencing to assess the overall structure of gut microbes in the CN, HFD, and NOB-H groups, the dilution curves of each sample gradually flattened, indicating the reliability of the sequencing results (Figure 4A).

The diversity of gut microbes in HFD-induced MAFLD mice was reduced compared to that of the CN group. After NOB-H intervention (Figure 4B), the microbial diversity was improved. Analysis of similarity (ANOSIM) using Bray–Curtis distance showed significant differences in beta diversity among the three groups (*r* = 0.7493, *p* = 0.0011, Figure 4C). Nonmetric multidimensional scaling (NMDS) analysis based on OTU levels revealed substantial alterations in gut microbiota composition after HFD and NOB-H interventions (Figure 4D).

Linear discriminant analysis effect size (LEfSe) with an LDA threshold ≥ 4 was utilized to identify representative species with significant differences among the groups (Figure 4E,F). *Bacteroidia*, *Dubosiella*, *Desulfovibrio*, and *Alloprevotella* in the CN group had a substantial impact on intergroup differences. *Allobaculum*, *Enterobacteriaceae*, and *Lachnoclostridium* had a greater effect on differences between groups, while *Clostridia*, *Parasutterella*, and *Blautia* played a more significant role in intergroup differences in the NOB-H group.

*Firmicutes*, *Verrucomicrobia*, *Bacteroidota*, *Actinomycetot*, and *Proteobacteria* were the major populations in the feces. Compared to the CN group, the HFD group exhibited increased microbial abundance in the phyla of *Firmicutes* and *Proteobacteria* and decreased in *Verrucomicrobiota*, *Bacteroidota*, and *Actinomycetot*. These alterations in microbial abundance were induced by the HFD proximity to CN group levels after NOB-H administration (Figure 4G). At the genus level (Figure 4H,I), the relative abundance of *Allobaculum* was significantly higher in the HFD group than in the CN group. NOB-H treatment led to a decrease in the relative abundance of *Allobaculum*. Previous studies have suggested that this bacterium is detrimental to gut health [27,28] and exhibits a significant negative correlation with *Akkermansia muciniphila*, which is a known probiotic genus for treating metabolic disorders [29]. NOB-H intervention reversed the HFD-induced decrease in *Akkermansia* and *Enterobacteriaceae* abundance and restored it, while *Enterobacteriaceae* is a major producer of endotoxins in the gut [30]. Furthermore, NOB-H intervention resulted in a significant increase in the intestinal abundance of several genera primarily involved in BA metabolism, such as *Clostridioides* [31], *Bacteroides* [32], *Blautia* [33], *Roseburia* [34], *Oscillibacter* [35], and *Enterococcus* [36]. These microbes facilitate the conversion of bound or primary BAs to unbound and secondary BAs through BSH production or participation in 7α-dehydroxylation [37]. This finding aligned with the previous results from BA metabolomics.

**Figure 5:**
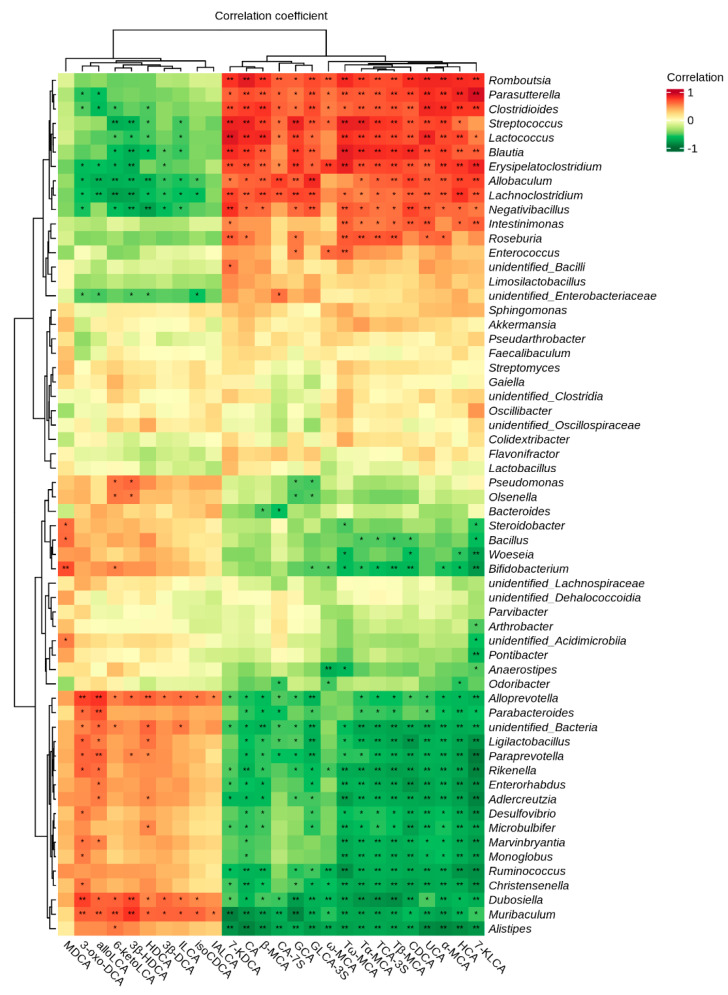
Correlation between differential BAs and differential gut microbiota. Values indicate Spearman’s correlation coefficients. * *p* < 0.05 and ** *p* < 0.01.

To further explore the relationship between changes in BA and gut microbiota, we conducted a Spearman rank correlation analysis between the 26 significant BA monomers and the top 60 genera of mouse gut microbial abundance. 44 kinds of the top 60 genera of microbes exhibited significant negative or positive correlations with at least one kind of BA (Figure 5).

For instance, the genera *Romboutsia*, *Paraprevotella*, *Clostridioides*, *Blautia*, *Erysipelatoclostridium*, and *Lachnoclostridium* exhibited positive correlations with various BAs including HCA, α-MCA, UCA, CDCA, TCDCA, Tβ-MCA, TCA-3S, Tω-MCA, Tα-MCA, ω-MCA, and 7-KDCA, while genera, such as *Alistipes*, *Muribaculum*, *Dubosiella*, and *Paraprevotella*, showed negative correlations with these BAs. The 3-oxo-DCA, 3β-HDCA, 3β-DCA, and 6-ketoLCA showed positive correlations with genera, such as *Muribaculum* and *Dubosiella*, but negative correlations with genera, such as *Lachnoclostridium*, *Erysipelatoclostridium*, and *Blautia*, revealing the significance of gut microbes in BA metabolism and its crucial role in the NOB-mediated attenuation of MAFLD.

### 2.7. NOB Reduces Liver Lipid Deposition by Regulating the Key Genes in BA Metabolism

The *FXR* plays a crucial role in maintaining BA homeostasis and regulating glucose and lipid metabolism [38]. Given the substantial changes in BA metabolism observed in mice after NOB intervention, we subsequently investigated the effects of NOB on the *FXR*/*SHP* signaling pathway. The protein and mRNA expression levels of the *FXR*/*SHP* signaling pathway in mouse liver were quantitatively analyzed using Western blot and RT-qPCR analyses.

**Figure 6:**
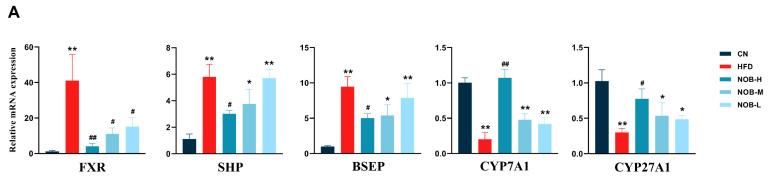

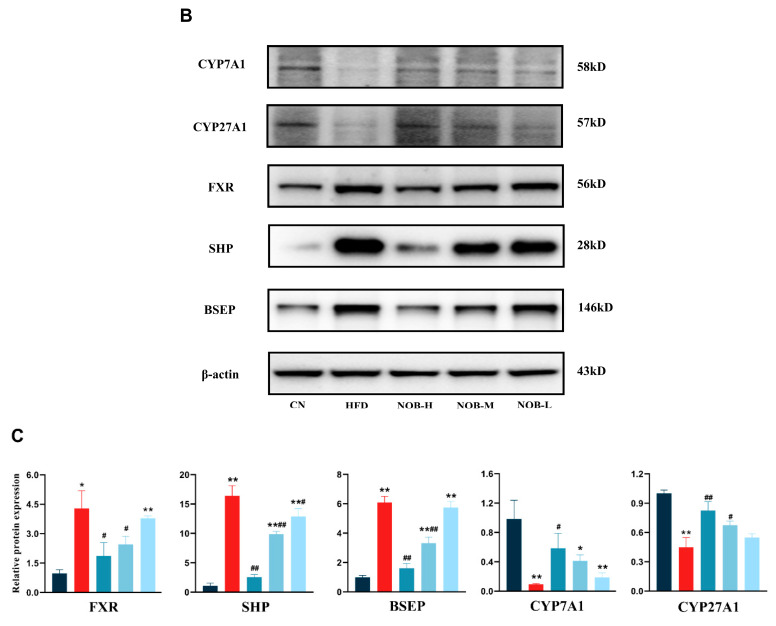
NOB alters BA metabolism-related mRNA and protein expression in the liver. (**A**) *FXR*, *SHP*, *BSEP*, *CYP7A1*, and *CYP27A1* relative mRNA expression levels in the liver. (**B**) Representative images of Western blotting for *FXR*, *SHP*, *BSEP*, *CYP7A1*, and *CYP27A1* in the liver. (**C**) Relative protein expression levels of *FXR*, *SHP*, *BSEP*, *CYP7A1*, and in the liver. * *p* < 0.05. ** *p* < 0.01 compared to the CN group; # *p* < 0.05, ## *p* < 0.01 compared to the HFD group (ANOVA, *n* = 3 in each group, mean ± SEM).

Compared to the CN group, the HFD group had significantly upregulated mRNA levels of *FXR*, *SHP*, and *BSEP*. NOB intervention, particularly at the medium and high doses, significantly reversed these changes. Additionally, both *CYP7A1*, the rate-limiting enzyme controlling the classical synthesis pathway of BA, and *CYP27A1*, the key enzyme involved in the alternative synthesis pathway of BA, exhibited reduced expression in the HFD group, but NOB intervention effectively reversed these changes (Figure 6A). Notably, the expression of *CYP7A1* surpassed that of the control group, suggesting that NOB increased BA synthesis via both the classical and alternative pathways. This observation aligned with the BA metabolomics results.

Compared to the CN group, the HFD group demonstrated an elevation in the protein expression of *FXR*, *SHP*, and *BSEP*, accompanied by a decrease in the expression of *CYP7A1* and *CYP27A1*. After NOB-H treatment, there was a decrease in the protein expression of *FXR*, *SHP*, and *BSEP*, alongside an increase in the protein expression of *CYP7A1* and *CYP27A1* (Figure 6B,C). These results suggest that in the HFD group, *FXR* and its downstream signals, *SHP* and *BSEP*, were highly activated, leading to the inhibition of cholesterol conversion to BA. However, the receipt of NOB-H facilitated the process of transforming cholesterol into BA.

## 3. Discussion

MAFLD, formerly known as NAFLD, was renamed and more precisely defined in 2020 [1]. This reclassification highlights the close relationship between MAFLD and metabolic abnormalities, drawing increased attention to explaining its pathogenesis and treatment from a metabolic aspect [39]. Although the exact mechanisms underlying MAFLD are still unclear now, extensive research has provided scientific evidence that the gut microbiome plays a significant role in its development [40,41,42,43]. Dysregulation of the gut ecosystem has emerged as a critical contributor to the onset and progression of MAFLD [44]. Prebiotics and probiotics have been considered potential therapies for MAFLD [45]. Natural products, particularly flavonoids [46,47], have shown potential in the treatment of MAFLD due to their powerful prebiotic properties [48].

NOB, a polymethoxyflavone derived from citrus, has previously demonstrated beneficial effects on lipid metabolism and hepatoprotection [49,50]. However, most of these reports solely focus on the association between NOB resistance to liver fat accumulation and gut microbiota without elucidating specific functional pathways. In the present study, we utilized an HFD-induced MAFLD model to investigate the benefits of NOB. We found that NOB treatment effectively slowed the progression of MAFLD by reducing serum lipid levels, blood glucose levels, LPS levels, and hepatic IL-1β and TNF-α levels induced by the HFD, consequently reducing lipid deposition. Both H&E staining and Oil Red O staining demonstrated that NOB improved liver pathological changes. Through further exploration of mechanisms, we discovered that NOB facilitates the transformation of cholesterol into primary BAs in the liver. Additionally, NOB enhances BA metabolism and excretion by increasing the abundance of BSH-producing gut bacteria. Several studies have suggested that high levels of BSH activity can lead to a significant proportion of unconjugated BAs, potentially causing lipid malabsorption and resulting in steatorrhea in the host. This observation aligns with our findings, as mice in the NOB-H group demonstrated increased food intake [51,52]. In this way, NOB facilitates the transformation of cholesterol and decreases lipid absorption, ultimately resulting in a positive impact on MAFLD.

BA are amphiphilic end products generated by cholesterol catabolism in the liver, playing a pivotal role in cholesterol metabolism, lipid uptake, and transport [53]. Given their effective enterohepatic circulation and physiological functions, the concentration and composition of BA influence lipid and glucose homeostasis and are strongly associated with MAFLD [54]. In the present study, targeted metabolomics indicated a significant increase in the total BA pool size and a reduction in hepatic lipid deposition in the NOB group compared to the HFD group, which indicated that the biosynthesis and accumulation of BA in the mouse liver were promoted. This observation was further supported by the substantial upregulation of hepatic levels of *CYP7A1* and *CYP27A1*, which are key enzymes controlling the major BA synthesis pathway. As a result of increased BA excretion, the liver increases cholesterol breakdown to produce BA to maintain BA pool homeostasis, ultimately reducing hepatic cholesterol accumulation [55]. Additionally, the composition of hydrophobic BA, such as CA, was decreased in the intestine, and the composition of hydrophilic BA, such as MCA, HCA, and HDCA, was significantly increased in the intestine, which may lead to the inhibition of lipid absorption and transport in the intestine [56].

The close interplay between the gut microbiota and BA is pivotal in regulating host glucose, cholesterol, and lipid metabolism [57]. 16S rRNA sequencing revealed significant improvements in the diversity of intestinal contents and the abundance of intestinal bacterial composition in MAFLD mice after NOB treatment. The shift in the ratio of primary to secondary BAs and conjugated to unconjugated BAs in feces was markedly associated with the prebiotic properties of NOB. Specifically, NOB effectively enhanced the abundance of genera capable of producing BSH and microbes involved in BA metabolism, such as *Clostridioides*, *Bacteroides*, *Blautia*, *Roseburia*, *Oscillibacter*, and *Enterococcus* [58]. This change resulted in increased catabolism of conjugated BAs into less soluble unconjugated BAs, which are more hydrophobic and more easily excreted with feces [51,59]. Consequently, reduced reabsorption led to enhanced synthesis of BA. Additionally, BSH activity disrupts the formation of BA micelles and the uptake of cholesterol and lipids [52]. NOB effectively decreased lipid uptake and increased cholesterol excretion by these mechanisms.

Previous research has shown that hepatic *FXR* activation upregulates *SHP*, leading to the inhibition of BA synthesis [60,61]. In addition, increased expression of *CYP7A1* promotes BA biosynthesis in mice fed a high-cholesterol diet, which ultimately reduces hepatic cholesterol accumulation [62]. In the present study, the NOB-enriched diet significantly reduced the protein and mRNA expression of hepatic *FXR*, *SHP*, and *BSEP* in MAFLD mice, and it significantly elevated the protein and mRNA expression of downstream genes, including *CYP7A1* and *CYP27A1*, corroborating our hypothesis regarding the regulatory effects of NOB on MAFLD.

## 4. Materials and Methods

### 4.1. Reagents and Chemicals

NOB (purity ≥ 98% determined by HPLC) was obtained from Tautochem (RM-0010, Shanghai, China). Neutral Buffered Formalin Fixative was purchased from Solarbio (G2162, Beijing, China), Hematoxylin was purchased from Servicebio (G1004, Wuhan, China). Eosin and Oil Red O were obtained from Bomeibio (YE2080, YO7512, Hefei, China), QIAamp DNAstool kits were obtained from Qiagen (51504, Hilden, Germany), TruSeq^®^ DNA PCR-Free Sample Preparation Kit was obtained from Illumina (20015962, San Diego, CA, USA), MolPure Cell/Tissue Total RNA Kit was obtained from Yeasen Biotech (19221ES60, Shanghai, China), PrimeScript RT reagent Kit was obtained from Takara (RR037A, Dalian, China), Cell Lysis Buffer and BCA Protein Assay were purchased from Beyotime Biotechnology (P0013, P0009, Beijing, China), Protease Inhibitor and Phosphatase Inhibitor were obtained from Biosharp (BL612A, BL615A, Hefei, China), and High-Fat Diets providing 60% of energy from fat were acquired from Xiaoshuyoutai (D12492, Beijing, China). AST (aspartate aminotransferase, C010-2-1), ALT (alanine aminotransferase, C009-2-1), TG (triglyceride, A110-1-1), TC (total cholesterol, A111-1-1), NEFA (nonesterified fatty acid, A042-2-1), HDL (high-density lipoprotein, A112-1-1), LDL (low-density lipoprotein, A113-1-1) assay kit, as well as LPS (lipopolysaccharide, H255-1-2), TNF-α (tumor necrosis factor-α, H052-1-2), IL-1β (Interleukin-1β, H002-1-2), and INS (Insulin, H203-1-2) ELISA kits were purchased from Nanjing Jiancheng Bioengineering Institute (Nanjing, China), Primary antibodies for *FXR* (abs122163), *CYP7A1* (abs132991), CYP27A-1 (abs125464), *BSEP* (abs134459), and *SHP* (abs136168) were procured from Absin, and goat anti-rabbit IgG (H + L) HRP was purchased from Affbiotech (S0001, Nanjing, China).

### 4.2. Animal Experimentation

The animal experiment protocol was approved by the Experimental Animal Ethics Committee of the Sichuan Academy of Traditional Chinese Medicine (approval number: SYLL (2022)-047). Five-week-old male C57BL/6J mice were obtained from Beijing HFK bioscience Co. Ltd. with an Animal Qualification Certificate (No.: SCXK (Beijing) 2019-0008). The mice were housed in the Animal Experimental Centre of the Sichuan Academy of Traditional Chinese Medicine. The mice were kept under controlled conditions with a relative humidity of 50~55%, a temperature of 22 ± 2 °C, unrestricted access to food and water, and a 12-h light-dark cycle. After a one-week acclimation period, the mice were randomly divided into the following five groups: control (CN), HFD, NOB-H, NOB- M, and NOB-L (*n* = 8 per group). The CN group was fed a normal diet and received a 0.5% CMC-Na solution, while the HFD group was fed a high-fat diet (HFD, 60% fat for energy) and received a 0.5% CMC-Na solution. Table S1 in the Supplementary Materials shows the composition and energy content of both the normal diet and HFD. The other three groups were fed a HFD and received different doses of NOB suspended in 0.5% CMC-Na as follows: low NOB dose (10 mg/kg, NOB-L), medium NOB dose (50 mg/kg, NOB-M), and high NOB dose (100 mg/kg, NOB-H). The NOB doses were determined based on prior research [49]. Both the 0.5% CMC-Na and NOB solutions were administered via daily oral gavage at a dose of 0.1 mL/kg of body weight.

MAFLD was induced following established research protocols [63]. The experiment lasted for 12 weeks, during which body weight and food consumption were monitored and recorded weekly. Additionally, Humane endpoints were established to manage any serious adverse reactions. At the end of the 12th week, fasting blood glucose (FBG) levels were measured using a Roche glucometer (ACCU-CHEK Performa, Leverkusen, Germany). Subsequently, the mice were euthanized by cervical dislocation after anesthesia with sodium pentobarbital following an overnight fast. Blood samples were collected and centrifuged (4 °C, 5000 rpm, 10 min) to obtain serum samples. Liver tissue, isolated intestinal tissue, and feces samples were collected with liquid nitrogen and then stored at −80 °C for subsequent analysis.

### 4.3. Biochemical Analysis and Histological Analysis

The collection and processing methods for mouse serum and liver tissue were adapted from previous studies [24]. The serum levels of TC, TG, NEFA, HDL, LDL, ALT, AST, LPS, and INS, as well as the hepatic levels of TC, IL-1Β, and TNF-α were detected using biochemistry kits, following the manufacturers’ specifications. Portions of liver and ileum tissues were immersed in a neutral formaldehyde solution and subsequently processed into standard paraffin sections with a thickness of 4 μm. The sections were dehydrated using a fully automated dehydrator. Hematoxylin and Eosin (H&E) staining was conducted to assess the histopathology of mouse liver and ileum tissues. Part of the liver tissue was frozen and embedded in OCT (optimal cutting temperature) compound and sliced. Subsequently, Oil Red O staining was employed to evaluate lipid accumulation in hepatocytes and determine the lipid droplet area. Image analysis was performed using the Image Pro Plus 6.0 image analysis system. All sections were visualized under a microscope, and images were captured using a Pannoramic 250 digital section scanner (3D HISTECH, Budapest, Hungary).

### 4.4. Metabolomics Analysis of BA

The Feces samples were stored at −80 °C after collection. BA targeted metabolomics analysis of the feces samples was performed by Metware Biotech Co., Ltd. (Wuhan, China). Feces samples (20 mg) were processed using a ball mill, and the ground samples were subjected to extraction with 200 μL of 20% methanol acetonitrile. To facilitate quantitative analysis, 10 μL of an internal standard mixed solution (1 μg/mL) was added to extract BA. Subsequently, the mixture was placed at −20 °C for 10 min to precipitate proteins. The samples were then centrifuged at 4 °C (12,000 rpm for 10 min), and the supernatant was transferred and collected. For the subsequent UPLC-MS analysis, the extracts were reconstituted after evaporation to dryness in 100 μL of 50% methanol (*v*/*v*). The details regarding the LC–MS procedures and data analysis are provided in the Supporting Information.

### 4.5. Feces Microbiota 16S rRNA Analysis

The feces samples were stored at −80 °C after collection. 16S rRNA analysis of the feces samples was performed by Metware Biotech Co., Ltd. (Wuhan, China). The genomic DNA extraction was performed using QIAamp DNA stool kits, and then the purity and concentration of the DNA were detected by agarose gel electrophoresis. The DNA sample was diluted with sterile water to a final concentration of 1 ng/µL. The V3–V4 region of the bacterial 16S ribosomal RNA gene was amplified by PCR. Then, 2% agarose gel was used for the detection of PCR products via electrophoresis, and equal samples were mixed according to the PCR product concentration. The target bands were recovered using a gel recovery kit provided by Qiagen. The library was constructed using TruSeq^®^ DNA PCR-Free Sample Preparation Kit. The constructed library was quantified by Qubit and q-PCR. After the library was qualified, NovaSeq6000 was used for online sequencing. Use the Uparse software (UPARSE v7.0.1001, http://www.drive5.com/uparse/; accessed on 1 October 2023) to cluster all the effective tags of all the samples. The sequences are clustered with 97% identity, becoming OTUs (Operational Taxonomic Units). Further noise reduction using the Deblur plugin (v1.1.1) for the QIIME 2 (https://qiime2.org/; accessed on 1 October 2023) platform was applied to generate an ASV (Amplicon Sequence Variant). The data analysis was performed using the free online platform of Metware Cloud Platform (https://cloud.metware.cn/; accessed on 1 October 2023). Alpha diversity and beta diversity analyses were performed using R software (v4.2.0), and functional annotation of microbes was conducted using Tax4 Fun2 (v1.1.5).

### 4.6. mRNA Expression Analysis

Portions of liver samples were stored at −80 °C after collection, abd mRNA Expression Analysis of the liver samples was performed by the Lilai Biomedicine Experiment Center (Chengdu, China). Total liver RNA was isolated with the help of the MolPure Cell/Tissue Total RNA Kit, and cDNA was reverse transcribed using the PrimeScript RT reagent Kit with a gDNA eraser. Quantitative reverse transcription PCR (RT-qPCR) analysis was conducted on the QuantStudio™ 3 (Thermo, Waltham, MA, USA). The relative mRNA expression of the target gene was calculated by the 2^−ΔΔCt^ method with normalization of β-actin. All primers were sourced from the National Centre for Biotechnology Information (NCBI) database, and they were synthesized and purified by Sangon Biotech (Shanghai, China). Primer sequences are listed in Table 1.

### 4.7. Western Blot

Total protein extraction was performed using Cell lysis buffer from frozen liver tissues, followed by centrifugation at 12,000 rpm for 10 min. The supernatants were collected and normalized with the BCA quantification kit. The protein was denatured at 95 °C for 15 min and subjected to electrophoresis on an SDS–PAGE gel. The proteins were transferred onto PVDF membranes, which were blocked with 5% skim milk diluted in TBST Buffer for 2 h. Subsequently, the membranes were incubated at 4 °C on a shaker overnight with the following primary antibodies: *BSEP* (1:2000), *CYP7A1* (1:2000), *CYP27A1* (1:2000), *FXR* (1:2000), *SHP* (1:2000), and *β-actin* (1:50,000). The membranes were then washed three times with TBST and incubated with secondary antibodies for 2 h. The protein bands were visualized using an ECL solution and a Tanon Fluorescence Image Analysis System Software V2.0. *β-Actin* served as the internal reference protein. The relative expression of the protein bands was analysed using Gel-Pro analyzer 4.0 software.

### 4.8. Statistical Analysis

All data are presented as the mean ± SEM. One-way ANOVA test or the Kruskal–Wallis (K–W test) test was selected according to the normality and homogeneity of variance of the data, and one-way ANOVA followed by the LSD multiple-range test. *p* < 0.05 was considered statistically significant. The formula used to calculate the HOMA-IR index was [HOMA−IR=[fasting insulin (mU/L)×fasting blood glucose (mmol/L)]/22.5] [64]. Graphical representations were created using GraphPad Prism 9.5.1.

## 5. Conclusions

In conclusion, the present study highlights the potential of NOB to improve MAFLD. NOB promotes BA excretion while inhibiting intestinal lipid absorption. These effects are attributed to an increase in total BA production and a rise in the number of intestinal bile acid-producing bacteria. Indeed, this study has not definitively determined whether the role of gut flora is crucial. Specifically, it remains unclear whether NOB affects flora balance by inducing an increase in bile acid synthesis or promotes bile acid metabolism by regulating flora balance. In future research, we plan to identify the relationship between these factors by utilizing germ-free or pseudo-germ-free animals. The treatment of MAFLD has been a complex issue for many years, and the present study provides a unique perspective and strategy for further advancements in this area.

## Figures and Tables

**Table 1:** Primer sequences for RT-qPCR.

Gene	Sequence 5′ → 3′ (Forward)	Sequence 3′ → 5′ (Forward)
*CYP7A1*	GTGATGTTTGAAGCCGGATATC	TTTATGTGCGGTCTTGAACAAG
*CYP27A1*	GACCATCGGCACCTTTCCTGAG	GGCACCACACCAGTCACTTCC1
*FXR*	GCAACCAGTCATGTACAGATTC	TTATTGAAAATCTCCGCCGAAC
*SHP*	GTCCGACTATTCTGTATGCACT	CTACTGTCTTGGCTAGGACATC
*BSEP*	GTGTCTACTTCATGCTTGTGAC	GAGACTTAGATCGTTGACGGAT
*β* *-actin*	CTACCTCATGAAGATCCTGACC	CACAGCTTCTCTTTGATGTCAC

## Data Availability

The data are available from the corresponding author upon reasonable request.

## Supplementary Materials

The following supporting information can be downloaded at: https://www.mdpi.com/article/10.3390/molecules29050976/s1, Supporting Information: The composition and energy content of both the normal and HFD diet, Details of PCR amplification and analysis of 16S rRNA, Conditions and procedures of BA targeted metabolomics analysis, total ion flow (TIC) and coefficient of variation (CV) plots for quality control analysis.
