# Supplementary material for: Involvement of Bile Acid Metabolism and Gut Microbiota in the Amelioration of Experimental Metabolism-Associated Fatty Liver Disease by Nobiletin
Source: Molecules. 2024 Feb 23;29(5):976. doi: 10.3390/molecules29050976 (PMC10935266; doi:10.3390/molecules29050976)

Nobiletin mitigates MAFLD by regulating intestinal flora and bile acid metabolism--Supplementary Information

Table S1. The composition and energy content of normal diet and HFD

products	normal diet (GB12924-1022)		HFD (D12492)	
	gm%	Kcal%	gm%	Kcal%
Protein	19.2	22.47	26.2	20
Carbohydrate	67.3	65.42	26.3	20
Fat	4.3	12.11	34.9	60
Total		100		100
Kcal/gm	3.42		5.24	
Ingredient content - normal diet (%)			Ingredient content- HFD (%)	
Crude protein	18.34		Casein, 80 Mesh	25.84
Crude fat	4.6		L-Cystine	0.39
Crude ash	4.0		Maltodextrin 10	16.15
Crude fiber	6.3		Sucrose	8.89
Water content	8.8		Cellulose, BW200	6.46
Calcium	1.19		Soybean Oil	3.23
Phosphorus	0.87		Lard*	31.66
Nitrogen-Free Extract	55.9		Mineral Mix S10026	1.29
/	/		DiCalcium Phosphate	1.68
/	/		Calcium Carbonate	0.71
/	/		Potassium Citrate, 1 H20	2.13
/	/		Vitamin Mix V1001	1.29
/	/		Choline Bitartrate	0.26
/	/		FD&C Blue Dye #1	0.01

Determination of microbial abundance in the feces

1、Sequencing

1.1 Extraction of genome DNA

Total genome DNA from samples was extracted using CTAB method. DNA concentration and purity was monitored on 1% agarose gels. According to the concentration, DNA was diluted to 1ng/ffL using sterile water.

1.2 Amplicon Generation

16S rRNA / 18S rRNA / ITS genes of distinct regions (16S V4/16S V3/16S V3-V4/16S V4-V5, 18S V4/18S V9, ITS1/ITS2, Arc V4) were amplified used specific primer (e.g. 16S V4: 515F- 806R, 18S V4: 528F-706R, 18S V9: 1380F-1510R, et.al.) with the barcode. All PCR reactions were carried out with 15 ffL of Phusion® High -Fidelity PCR Master Mix (New England Biolabs); 2 ffM of forward and reverse primers, and about 10 ng template

DNA. Thermal cycling consisted of initial denaturation at 98 °C for 1 min, followed by 30 cycles of denaturation at 98 °C for 10 s, annealing at 50°C for 30 s, and elongation at 72°C for 30 s. Finally 72°C for 5 min.

1.3 PCR Products quantification and qualification

Mix same volume of 1X loading buffer (contained SYB green) with PCR products and operate electrophoresis on 2% agarose gel for detection. PCR products was mixed in equidensity ratios. Then, mixture PCR products was purified with Qiagen Gel Extraction Kit (Qiagen,Germany).

1.4 Library preparation and sequencing

Sequencing libraries were generated usingTruSeq® DNA PCR-Free Sample Preparation Kit (Illumina, USA) following manufacturer's recommendations and index codes were added.The library quality was assessed on the Qubit@ 2.0 Fluorometer (Thermo Scientific) and Agilent Bioanalyzer 2100 system. At last, the library was sequenced on an Illumina NovaSeq platform and 250 bp paired-end reads were generated.

2 Data analysis

2.1 Paired-end reads assembly and quality control

2.1.1 Data split

Paired-end reads was assigned to samples based on their unique barcode and truncated by cutting off the barcode and primer sequence.

2.1.2 Data Filtration

Quality filtering on the raw tags were performed under specific filtering conditions to obtain the high-quality clean tags according to the fastp (v0.22.0, https://github.com/OpenGene/fastp).

2.1.3 Sequence assembly

Paired-end reads were merged using FLASH (v1.2.11, http://ccb.jhu.edu/software/FLASH/)(Magoc and Salzberg 2011), a very fast and accurate analysis tool, which was designed to merge paired-end reads when at least some of the reads overlap the read generated from the opposite end of the same DNA fragment.

2.1.4 Chimera removal

The tags were compared with the reference database (Silva database (16S/18S) https://www.arb-silva.de/; Unite Database(ITS), https://unite.ut.ee/) using UCHIME Algorithm (http://www.drive5.com/usearch/manual/uchime_algo.html) (Edgar et al. 2011) to detect chimera sequences, and then the chimera sequences were removed (Haas et al. 2011). Then the Effective Tags finally obtained.

2.2 OTU/ASV and Species annotation

2.2.1 OTU Clustering

Sequences analysis were performed by Uparse software (Uparse v7.0.1001, http://drive5.com/uparse/) (Edgar 2013). Sequences with ff97% similarity were assigned to the same OTUs. Representative sequence for each OTU was screened for further annotation.

2.2.2 ASV denoising

Amplicon sequence variant (ASV) were analysised by Deblur, which uses error profiles to obtain putative error-free sequences from Illumina MiSeq and HiSeq sequencing platforms.

2.2.3 Species annotation

• 16S: For each representative sequence, the Silva Database (http://www.arb-silva.de/) (Quast et al. 2012) was used based on Mothur algorithm to annotate taxonomic information.

• 18S: For each representative sequence, the Silva Database (http://www.arb-silva.de/) (Quast et al. 2012) was used based on RDP classifier algorithm to annotate taxonomicinformation.

• ITS: For each representative sequence, the Unite Database (https://unite.ut.ee/) (Kõljalg et al. 2013) was used based on blast algorithm to annotate taxonomic information.

2.2.4 Phylogenetic relationship Construction

In order to study phylogenetic relationship of different OTUs, and the difference of the dominant species in different samples (groups), multiple sequence alignment were conducted using the MAFFT (v7.490, https://mafft.cbrc.jp/alignment/software/) (Katoh et al. 2002).

2.2.5 Data Normalization

OTUs abundance information were normalized using a standard of sequence number corresponding to the sample with the least sequences. Subsequent analysis of alpha diversity and beta diversity were all performed basing on this output normalized data.

3 Alpha Diversity

Alpha diversity is applied in analyzing complexity of species diversity for a sample through 6 indices, including Observed-species, Chao1, Shannon, Simpson, ACE, Good-coverage. All this indices in our samples were calculated with QIIME and displayed with R software (Version 4.1.2).

Two indices were selected to identify Community richness:

• Chao -the Chao1 estimator (http://www.mothur.org/wiki/Chao);

• ACE -the ACE estimator (http://www.mothur.org/wiki/Ace);

Two indices were used to identify Community diversity:

• Shannon - the Shannon index (http://www.mothur.org/wiki/Shannon);

• Simpson - the Simpson index (http://www.mothur.org/wiki/Simpson);

One indice to characterized Sequencing depth:

• Coverage - the Good's coverage (http://www.mothur.org/wiki/Coverage)

4 Beta Diversity

Beta diversity analysis was used to evaluate differences of samples in species complexity, Beta diversity on both weighted and unweighted unifrac were calculated by QIIME software.

Cluster analysis was preceded by principal component analysis (PCA), which was applied to reduce the dimension of the original variables using the stats package and ggplot2 package in R software.Principal Coordinate Analysis (PCoA) was performed to get principal coordinates and visualize from complex, multidimensional data. A distance matrix of weighted or unweighted unifrac among samples obtained before was transformed to a new set of orthogonal axes, by which the maximum variation factor is demonstrated by first principal coordinate, and the

second maximum one by the second principal coordinate, and so on. PCoA analysis was displayed by stats package and ggplot2 package in R software.Unweighted Pair-group Method with Arithmetic Means (UPGMA) Clustering was performed as a type of hierarchical clustering method to interpret the distance matrix using average linkage and was conducted by QIIME software

Determination of Bile Acid Concentrations in Colon Contents

1、Methods of Experimental

1.1 Chemicals and reagents

HPLC-grade acetonitrile (ACN) and methanol (MeOH) were purchased from Merck (Darmstadt, Germany). MilliQ water (Millipore, Bradford, USA) was used in all experiments. All of the standards were purchased from CNW (Shanghai, China) and IsoReag (Shanghai, China). Acetic acid and ammonium acetate were bought from Sigma-Aldrich (St. Louis, MO, USA). The stock solutions of standards were prepared at the concentration of 1 mg/mL in MeOH. All stock solutions were stored at -20°C. The stock solutions were diluted with MeOH to working solutions before analysis.

1.2 Sample preparation and extraction

Samples (20 mg) were extracted with 200 μL methanol/acetonitrile(v/v=2:8) after the samples grinded with ball mill. 10 μL internal standard mixed solution (1 μg/mL) was added into the extract as internal standards (IS) for the quantication. Put the samples at -20°C for 10 min to precipitated protein. Then centrifugation for 10 min (12000 r/min, and 4°C), the supernatant was transferred to the sample bottle for further LC-MS analysis.

1.3 HPLC Conditions

The sample extracts were analyzed using an LC-ESI-MS/MS system (UHPLC， ExionLC™ AD，https://sciex.com.cn/ ; MS，Applied Biosystems 6500 Triple Quadrupole, https://sciex.com.cn/). The analytical conditions were as follows, HPLC: column, Waters ACQUITY UPLC HSS T3 C18 (100 mm×2.1 mm i.d.，1.8 μm); solvent system, water with 0.01% acetic acid and 5mmol/L ammonium acetate (A), acetonitrile with 0.01% acetic acid (B); The gradient was optimized at 5% to 40%B in 0.5 min, then increased to 50% B in 4 min, then increased to 75% B in 3 min, and then 75% to 95% in 2.5min, washed with 95%B for 2 min ,finaly ramped back to 5% B (12-14 min); flow rate, 0.35 mL/min; temperature, 40°C; injection volume: 1 μL. The effluent was alternatively connected to an ESI-triple quadrupole-linear ion trap (QTRAP)-MS.

1.4 ESI-MS/MS Conditions

Linear ion trap (LIT) and triple quadrupole (QQQ) scans were acquired on a triple quadrupole-linear ion trap mass spectrometer (QTRAP), QTRAP® 6500+ LC-MS/MS System, equipped with an ESI Turbo Ion-Spray interface, operating in negative ion mode and controlled by Analyst 1.6.3 software (Sciex). The ESI source operation parameters were as follows: ion source, ESI-; source temperature 550 ℃; ion spray voltage (IS) -4500 V; curtain gas (CUR) was set at 35 psi, respectively. Bile acids were analyzed using scheduled multiple reaction monitoring (MRM). Data acquisitions were performed using Analyst 1.6.3 software (Sciex). Multiquant 3.0.3 software (Sciex) was used to quantify all metabolites. Mass spectrometer parameters including the

declustering potentials (DP) and collision energies (CE) for individual MRM transitions were done with further DP and CE optimization. A specific set of MRM transitions were monitored for each period according to the metabolites eluted within this period.

2、Methods of analysis

2.1 PCA

Unsupervised PCA (principal component analysis) was performed by statistics function prcomp within R (www.r-project.org). The data was unit variance scaled before unsupervised PCA.

2.2 Hierarchical Cluster Analysis and Pearson Correlation Coefficients

The HCA (hierarchical cluster analysis) results of samples and metabolites were presented as heatmaps with dendrograms, while pearson correlation coefficients (PCC) between samples were caculated by the cor function in R and presented as only heatmaps. Both HCA and PCC were carried out by R package pheatmap. For HCA, normalized signal intensities of metabolites (unit variance scaling) are visualized as a color spectrum.

2.3 Differential metabolites selected

Significantly regulated metabolites between groups were determined by VIP and absolute Log2FC (fold change). VIP values were extracted from OPLS-DA result, which also contain score plots and permutation plots, was generated using R package MetaboAnalystR. The data was log transform (log2) and mean centering before OPLS-DA. In order to avoid overfitting, a permutation test (200 permutations) was performed.

2.4KEGG annotation and enrichment analysis

Identified metabolites were annotated using KEGG compound database (http://www.kegg.jp/kegg/compound/), annotated metabolites were then mapped to KEGG Pathway database (http://www.kegg.jp/kegg/pathway.html). Pathways with significantly regulated metabolites mapped to were then fed into MSEA (metabolite sets enrichment analysis), their significance was determined by hypergeometric test's P-Values.

Bile acids standards.

	Abbreviation	Common Name
1	TLCA-3S	taurolithocholic acid-3-sulfate
2	DLCA	Dehydrolithocholic acid
3	IALCA	Isoallolithocholic acid
4	LCA	Lithocholic acid
5	ILCA	isolithocholic acid
6	23-DCA	Nor-Deoxycholic Acid
7	3-oxo-DCA	3-oxodeoxycholic acid
8	7-KLCA	7-ketolithocholic acid
9	12-KLCA	12-ketolithocholic acid
10	MDCA	murideoxycholic acid
11	DCA	Deoxycholic acid
12	IDCA	Isodeoxycholic acid

13	3β-DCA	3β-deoxycholic acid
14	3β-UDCA	3β-Ursodeoxycholic Acid
15	UDCA	Ursodeoxycholic acid
16	3β-HDCA	β-Hyodeoxycholic Acid
17	HDCA	Hyodeoxycholic acid
18	CDCA	Chenodeoxycholic acid
19	NCA	norcholic acid
20	DHCA	Dehydrocholic acid
21	7,12-DKLCA	7,12-diketolithocholic acid
22	6,7-DKLCA	6,7-diketolithocholic acid
23	3-oxo-CA	3-Oxocholic acid
24	12-oxo-CDCA	12-Oxochenodeoxycholic acid
25	7-KDCA	7-Ketodeoxycholic acid
26	ω-MCA	ω-muricholic acid
27	3β-CA	3β-Cholic Acid
28	CA	cholic acid
29	α-MCA	α-muricholic acid
30	HCA	hyocholic acid
31	UCA	Ursocholic acid
32	β-MCA	β-muricholic acid
33	GLCA	Glycolithocholic acid
34	GUDCA	Glycoursodeoxycholic acid
35	GCDCA	Glycochenodeoxycholic acid
36	GDCA	Glycodeoxycholic acid
37	LCA-3S	lithocholic acid-3-sulfate
38	GDHCA	Glycodehydrocholic acid
39	GHCA	Glycohyocholic acid
40	GCA	Glycocholic acid
41	TLCA	taurolithocholic acid
42	TDCA	Taurodeoxycholic acid
43	TUDCA	Tauroursodeoxycholic acid
44	TCDCA	Taurochenodeoxycholic acid
45	TDHCA	Taurodehydrocholic acid
46	GLCA-3S	glycolithocholic acid-3-sulfate
47	THCA	Taurohyocholic acid
48	Tβ-MCA	Tauro-β-muricholic acid
49	TCA	Taurocholic acid
50	CDCA-3Gln	Chenodeoxycholic acid-3-β-D-glucuronide
51	6-ketoLCA	5-β-Cholanic Acid-3α-ol-6-one
52	alloLCA	5α-CHOLANIC ACID-3α-OL
53	isoCDCA	IsochenodeoxycholicAcid
54	βGCA	3β-Glycocholic Acid
55	GHDCA	Glycohyodeoxycholic Acid
56	THDCA	TaurohyodeoxycholicAcid (sodium salt)

57	Tα-MCA	Tauro-α-muricholicAcid sodium salt
58	Tω-MCA	Tauro-ω-muricholicAcid sodium salt
59	CA-3S	Cholic Acid 3 Sulfate-Sodium Salt
60	CA-7S	cholic acid 7 sulfate
61	DCA-3-O-S	Deoxycholic Acid 3-O-Sulfate Disodium Salt
62	CDCA-3S	chenodeoxycholic acid-3-sulfate disodium salt
63	GCDCA-3S	Glycochenodeoxycholic-Acid 3 Sulfate Disodium Salt
64	GUDCA-3S	Glycoursodeoxycholic-Acid 3 Sulfate Sodium
65	TCA-3S	Taurocholic Acid 3 sulfate sodium salt

QC Sample total ion flow diagram (TIFD)

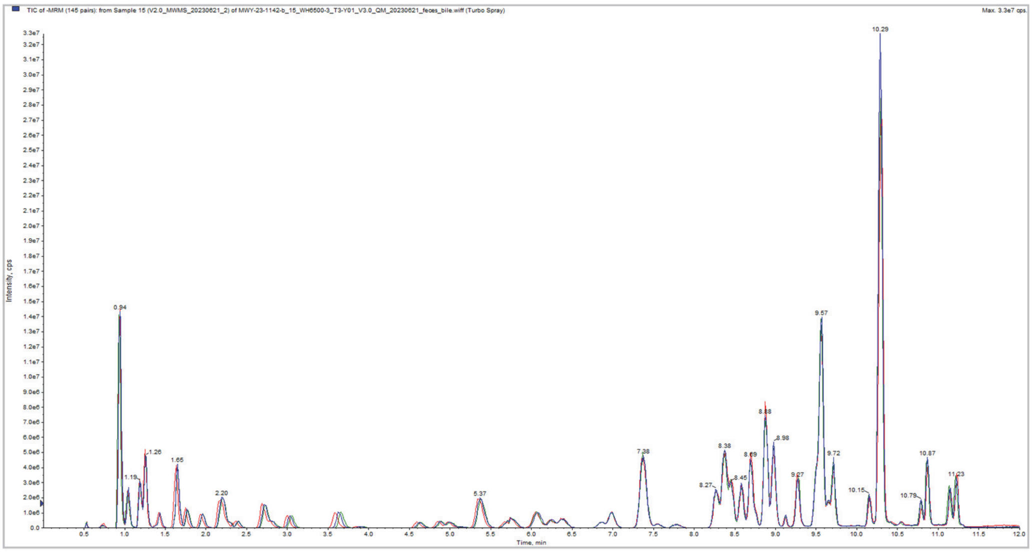

QC Sample Coefficient of Variation

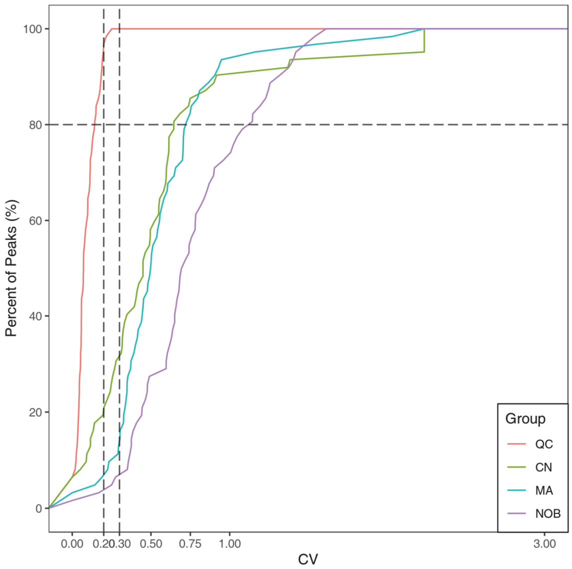

Supplement: Supplementary file 1 [file molecules-29-00976-s001.zip › molecules-2840915-supplementary.pdf]
